# Aptamers and antibodies: rivals or allies in cancer targeted therapy?

**DOI:** 10.37349/etat.2021.00035

**Published:** 2021-02-28

**Authors:** Lisa Agnello, Simona Camorani, Monica Fedele, Laura Cerchia

**Affiliations:** Institute of Experimental Endocrinology and Oncology “Gaetano Salvatore”, National Research Council (CNR), Via S. Pansini 5, 80131 Naples, Italy; University of Southampton, UK

**Keywords:** Aptamers, monoclonal antibodies, targeted therapy, theranostics, SELEX

## Abstract

The goal of an efficacious cancer therapy is to specifically target diseased cells at high accuracy while sparing normal, healthy cells. Over the past three decades, immunotherapy, based on the use of monoclonal antibodies (mAbs) directed against tumor-associated antigens, to inhibit their oncogenic function, or against immune checkpoints, to modulate specific T cell responses against cancer, has proven to be an important strategy for cancer therapy. Nevertheless, the number of mAbs approved for clinical use is still limited because of significant drawbacks to their applicability. Oligonucleotide aptamers, similarly to antibodies, form high-affinity bonds with their specific protein targets, thus representing an effective tool for active cancer targeting. Compared to antibodies, aptamers’ use as therapeutic agents benefits from their low size, low/no immunogenicity, simple synthesis and design flexibility for improving efficacy and stability. This review intends to highlight recently emerged applications of aptamers as recognition elements, from biomarker discovery to targeted drug delivery and targeted treatment, showing aptamers’ potential to work in conjunction with antibodies for attacking cancer from multiple flanks.

## Introduction

Cancer, often referred to as the “Pathology of the Century”, represents the second leading cause of death worldwide, affecting millions of people each year [1, 2]. Cytotoxic chemotherapy and radiotherapy are the main treatment options for many patients as neoadjuvant or adjuvant to surgery, however their success is often limited by several drawbacks, in part due to the lack of selectivity for tumor cells.

Typically, conventional chemotherapeutics target highly proliferating cells, resulting unable to discriminate cancer cells from rapidly dividing healthy cells and thus producing severe side effects to normal tissues. This strongly reduces the amount of drug that can be systemically administered and, together with pharmacokinetic effects, hampers its effectiveness to the tumor site. Moreover, the development of multiple chemoresistance mechanisms, including decreased uptake or increased efflux of the drug, low activation of prodrug or higher drug inactivation, DNA damage repair, mutations of drug targets, occurrence of compensatory signaling pathways and evasion of cell death, represents the major cause of tumor relapse and metastasis, hampering the clinical outcome [3–5]. Also, a paradoxical effect of chemotherapy has been evidenced by which its therapeutic efficacy on the primary tumor can be balanced by the stimulation of responses capable of inducing epithelial-mesenchymal transition, stem phenotype and other protective mechanisms, which in turn modify tumor microenvironment (TME), causing detrimental effects on tumor relapse and promotion of metastases [6]. Healthy secretome is composed by a great variety of factors and contributes to maintain homeostasis in the microenvironment. Chemotherapy can induce alterations in the tumor cell secretome composition, shaping it in a collection of protumorigenic factors that promote the formation of an immunosuppressive TME [7]. Therefore, TME can become a shield for cancer cells, protecting them from drugs insults and contributing to the development of chemoresistance.

Radiotherapy is equally subject to mechanisms of resistance and has profound effects on TME. Interestingly, radiotherapy, in addition to attacking cancer cells by inducing DNA damage, modulates diverse components of the TME, producing anti-tumor immunomodulatory effects [8]. However, radiotherapy, as chemotherapy, encounters the bottlenecks caused by increased resistance to treatment by multiple mechanisms including genes mutation or their aberrant expression, epigenetic alterations, cancer stem cells existence, the over activation of same signaling pathways and TME alteration [9]. To overcome the limitations of conventional chemotherapy and radiotherapy, a huge effort is devoted to identify cancer-specific biomarkers, usually proteins found mutated or abnormally expressed in cancer cells, which can enable effective targeted therapies that target cancer cells specifically, killing them or stopping their growth or spread, while sparing healthy cells [10]. Two main classes of targeting cancer agents are currently available for use in clinical practice, monoclonal antibodies (mAbs) and small-molecule inhibitors [11]. So far, Food and Drug Administration (FDA) has approved several agents for specific types of cancer for which established therapeutic targets have been identified [12–14]. As example, trastuzumab and pertuzumab (mAbs) on the one hand, and lapatinib and neratinib (small molecules) on the other, all targeting and interfering with the human epidermal growth factor receptor 2 (HER2), have shown to be beneficial for treating patients affected by a subtype of breast cancer overexpressing HER2 [15]. Furthermore, the opportunity to use drugs in combination is a viable strategy to overcome resistance to single-agent targeted therapy, which often occurs. Indeed, crosstalk between pathways and feedback loops, which are essential aspects of cell physiology, could modify therapeutic vulnerability in cancer cells. Combinations of two or more therapeutic agents can be more effective than monotherapy approach, because they work by different mechanisms in a characteristically synergistic or additive manner [16, 17].

Unfortunately, targetable proteins have not yet been identified for several human cancers that still lack targeted therapy, including heterogeneous triple-negative breast cancer (TNBC) and glioblastoma (GBM) [18, 19]. In search for efficacious strategies to both identify new actionable biomarkers and specifically target cancer cells, alternatively and/or in combination with existing drugs, oligonucleotide aptamers have sparked intense interest [20, 21].

## Aptamers: the chemical antibodies

**Figure 1.**
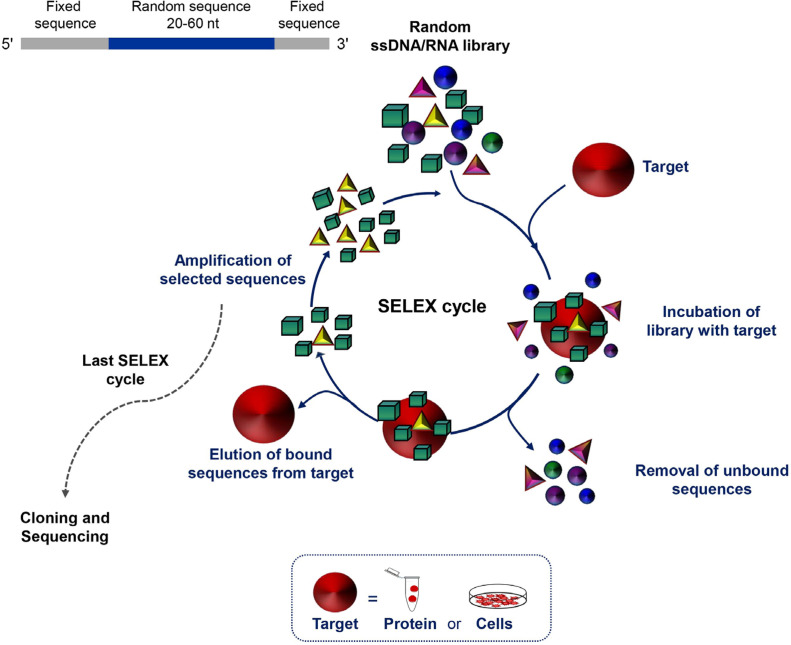
Schematic representation of the SELEX technology. SELEX is a multistep process starting with random libraries of single-stranded DNA (ssDNA) or RNA flanked by two fixed primer-binding sequences on both ends necessary for enzymatic amplification and *in vitro* transcription (in the case of a RNA library). It includes reiterated rounds (usually 8-20) of binding, partitioning and amplification (see text for details). nt: nucleotide

Aptamers, often termed “chemical antibodies”, are short, single-stranded DNA or RNA that assume three-dimensional structures to bind at high affinity to a specific target molecule. The targets of aptamers range from small chemicals to large cell-surface and transmembrane proteins even embedded in their natural environment, i.e. living cells, tissues or animals. Their easy and reproducible manufacture, combined with the nucleic acid composition that supports a plethora of chemical modifications, which in turn allow for different conjugation chemistries, makes aptamers suitable for molecular recognition-based applications, including cancer diagnosis and targeted therapy [22]. Aptamers are selected *in vitro* from large libraries of random sequence oligonucleotides using systematic evolution of ligands by exponential enrichment (SELEX) technology (Figure 1). SELEX consists of a multi-step process involving repeated cycles of: i) incubation of the library with the target; ii) partition of target-bound oligonucleotides from unbound sequences; iii) elution of the bound sequences frSom the target; and iv) amplification of the selected oligonucleotides. Individual sequences are identified by classical cloning of the final selected pool and/ or high-throughput sequencing and bioinformatics analyses, which allow to monitor the selection of aptamers during all SELEX rounds [23, 24]. Finally, the selected sequences are tested for binding affinity and specificity.

Introduced for the first time about thirty years ago [25–27], SELEX has aroused growing interest and has been successfully applied, to date, for the isolation of aptamers against proteins of biomedical interest for use as research agents, diagnostic agents, therapeutics, biosensor components and tools for biomarker and drug discovery [28]. In recent years, sophisticated SELEX protocols have been developed, optimizing the starting library and the separation techniques, in order to reduce the process times and the number of cycles for the selection of high efficacious aptamers endowed with the proper features required for their final application [29–31].

It is well known that cell surface protein biomarkers are of particular interest for cancer diagnosis and targeted therapy, and mAbs against antigens expressed on the surface of cancer cells represent a revolutionary therapeutic approach. Aptamers can be selected to target specific cell surface proteins, so they may have the same potential as mAbs immunotherapy.

To generate aptamers against a cell surface protein, a purified protein can be used as target for the selection, usually the peptide corresponding to the extracellular region (protein-SELEX approach) or, otherwise, live cells that overexpress the pre-identified target of interest (cell-SELEX approach). The latter approach allows selecting aptamers against protein targets as close as possible to their natural distributions and conformations. Protein-SELEX has the advantage of easy control of the enrichment conditions and takes usually shorter time than cell-SELEX. However, it often happens that aptamers generated against a recombinant protein do not recognize the protein in the endogenous environment [32]. For this reason, selection on whole cells [33] or strategies that alternate protein- and cell-SELEX [34, 35], even if trickier, are better suited for generating aptamers targeting cell-surface proteins especially for *in vivo* applications. Furthermore, an important application of the cell-SELEX consists in generating aptamers targeting cancer cells without prior knowledge of biomarkers present on the cell surface by alternating positive selection on target cells and counter-selection steps on off-target cells. At the end of the selection, a panel of aptamers will be identified that recognizes a surface molecular signature specific of the tumor cell type used for the positive selection. Importantly, at the end of cell-SELEX, the best binding aptamers are used as bait to identify their targets, thus proving to be an amazing tool for biomarker discovery. Using this approach, aptamers with cell-type discriminating properties were selected, which identify the surface molecular signatures of cancer cells associated with important behaviors, including resistance to treatment, tumorigenicity, stemness and metastatic potential [36–43]. Recently, by applying a differential cell-SELEX approach to TNBC cells, our group generated six novel nuclease-resistant RNA aptamers able to distinguish TNBC cells from both non-malignant and non-TNBC breast cancer cells, as well as to differentiate TNBC histological specimens in aptamer-based histochemical approaches [44]. Importantly, they are endowed with some biological activities, such as internalization within the target cells and anti-proliferative and anti-mammosphere activity [44]. The availability of a set of aptamers acting as high efficacious recognition probes for functional surface signatures of TNBC cells fulfills the great challenge of simultaneously targeting multiple protein targets.

Importantly, SELEX can also be realized in more complex cellular environments as cellular spheroids in three-dimensional cell culture systems [45], or even tissues [46] and whole-organisms *in vivo* [47–49]

## Unique properties of aptamers for targeted cancer therapy

Aptamers possess several unique features as recognition elements that rend them a valid alternative or complement to conventional antibodies in targeted cancer therapy (Table 1).

**Table 1.** Strengths and weaknesses of aptamers in comparison with antibodies

**Criteria**	**Aptamers**	**Antibodies**
Size	5–15 kDa	150–180 kDa
• Target accessibility	High	Low
• Minimal target size	60 Da	600 Da
• Tissue/tumor penetration	High	Low
• Clearance rate	Rapid	Slow
Basic composition	Nucleotides	Amino acids
• Resistance to harsh environment conditions (pH and temperature)	High	Low
• Shelf-life	Long	Limited
• Versatility to chemical	High	Limited
• Nuclease degradation	Sensitive; limited half-life *in vivo* (unmodified)	Resistant; long half-life *in vivo*
Therapeutic efficacy		
• Affinity and specificity	K_D_, nano/pico	K_D_, nano/pico
• Immunogenicity	Low/none	High
• Modulation of target activity	Yes	Yes
• Fc-mediated effector	No	Yes
Discovery		
• Time	*In vitro* SELEX, 2–8 weeks	*In vivo* biological process, 6
Production		
• Scale up	Easy	Hard
• Batch to batch variation	None	High

K_D_ values: dissociation constants

First, because of their production by chemical synthesis, the reproducibility of aptamers is very accurate and manufacture does not require the use of cell cultures and animals, which are instead required for the production of antibodies. In addition, with the increasing development of sophisticated technologies, the production of aptamers is becoming even more cost- and time-effective.

As a result of their complex tridimensional structures, aptamers like antibodies have high affinity and specificity for the target with K_D_ values typically in the low nanomolar-picomolar range. Like antibodies, aptamers can recognize with high specificity proteins of the same family, or even the same protein present in different conformational states [22, 50]. However, unlike antibodies, aptamers have a low size, usually from 5 to 15 kDa, which allows them to bind low molecular weight targets or some hidden binding domains that are inaccessible to larger antibodies. Further, their smaller size, 20- to 25-fold as compared with full-sized mAbs, is a crucial property that allows high tissue penetration, as needed for cancer treatment. Most importantly, aptamers support a huge variety of chemical modifications that are crucial to overcome undoubted limitations to their clinical applications, such as uncertain stability and rapid renal clearance. For instance, the susceptibility of aptamers to nuclease degradation is easily overcome by applying analogues of phosphate, ribose and bases [51], thus increasing their stability and half-life *in vivo* especially in the complex microenvironment that surrounds the tumor. Further, to increase their permanence in the circulation, bulky molecules are appended to the aptamer including polyethylene glycol (PEG) [52] or even antibodies [53], without affecting the high tumor penetration and target accessibility. More importantly, chemical modification allows reducing the potential *in vivo* toxicity and immunogenicity of aptamers, thus allowing for the safe administration of repeated doses that are precluded for antibodies, unless they are humanized or produced completely in humans (Table 1). An elegant example of an ad hoc chemical modification of aptamers was recently reported by Zhou et al. [54], which generated aptamers able to work only in hypoxic TMEs (conditional aptamers). Indeed, the addition of a PEG5000-azobenzene-NHS to the aptamer prevents binding to its target protein. When the conjugate reaches the tumor site, the hypoxic environment causes the release of the PEG-group from the aptamer that assumes a conformation competent for the recognition of the target. This strategy allows limiting the action of the aptamer exclusively to cancer cells, thus reducing toxicity towards healthy cells, in the case in which the target of the aptamer is not expressed only in tumors.

Finally, aptamers modifications allow to set up sophisticated combination therapies by chemically conjugating aptamers with other type of therapeutic molecules or drugs [22, 55], thus improving their effectiveness for clinical application.

## Applications of aptamers in cancer targeted therapy

**Figure 2.**
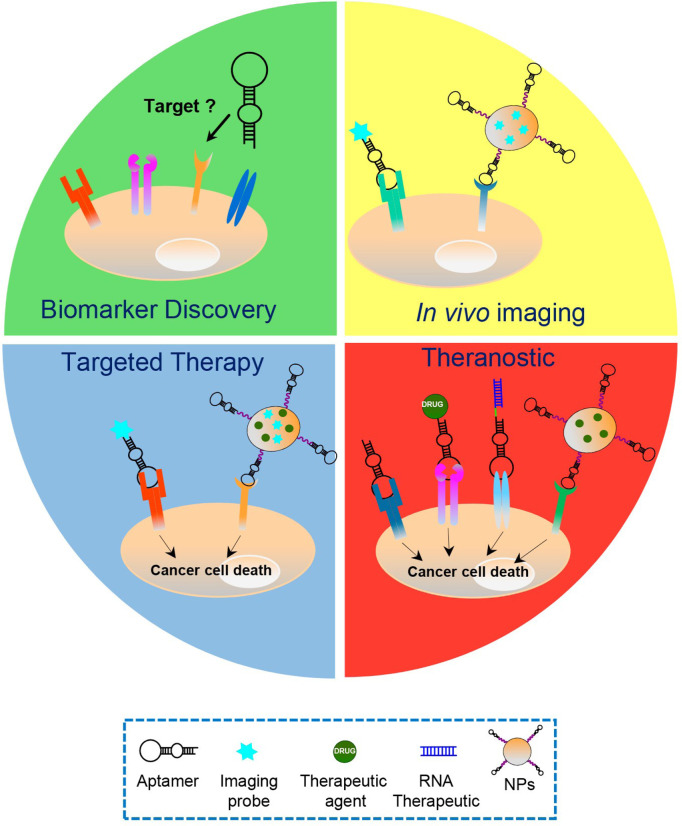
Aptamers’ applications in cancer treatment. Schematic representation of the various cancer applications of aptamers, including biomarker discovery, *in vivo* imaging, targeted therapy and theranostic approaches, which play key roles in the personalized cancer therapy. Aptamers have been used as: i) recognition agents for biomarkers discovery; ii) imaging agents upon conjugation to fluorescent probes, radionuclides or nanoparticles (NPs) functionalized with biomedical imaging agents; iii) stand-alone therapeutics or delivery agents for chemotherapeutics, RNA therapeutics and drug-loaded NPs; and iv) theranostic agents, upon conjugation to imaging agents (in case the aptamer itself has anti-tumor activities) or NPs loaded with both drug and imaging agents

For all the properties described above, cell-targeting aptamers have proven to be a valid alternative to mAbs in various cancer applications (Figure 2).

First, aptamers are foreseen as promising stand-alone therapeutic agents; indeed, by binding to their proper cancer-related target, they can interfere with its biological function, thus ultimately inhibiting tumor development and progression. They can act either as inhibitors of the target, competing with small molecules and protein ligands, as in the case of aptamers that inhibit human receptor tyrosine kinases [37, 56–61], or as activators, promoting the function of target receptors [62, 63]. In the latter case, usually the generation of a dimeric aptamer is able to bind to two receptor monomers, thus inducing dimerization and activation of the receptor. As an example of aptamers’ ability to equalize antibodies as targeted therapeutics, Mahlknecht et al. [64], compared the efficacy of the trimeric anti-HER2 DNA aptamer with a monoclonal antibody against the same receptor in reducing tumor growth in gastric cancer xenograft mice, showing the superior efficacy of the aptamer over the antibody. Recently, as demonstration of the striking therapeutic potential of aptamers, Yang et al. [65], has developed a new approach that harnesses the power of aptamer technology in cancer immunotherapy. They decorated the surface of natural killer (NK) cells with a CD30 aptamer by conjugating the aptamer to different lipophilic anchors intercalated into the cell membrane. Importantly, under the guidance of aptamers, NK cells specifically targeted CD30-expressing lymphoma cells, triggering cell apoptosis and death due to their innate killing capacity. This aptamer-based strategy, which conforms NK cells for cell-specific adaptive immunotherapy without genetic manipulation, opens a powerful new avenue for cancer-targeted immunotherapy.

The development of aptamer-based cancer therapy includes not only aptamers used as stand-alone agents, but also conjugated with other drugs. Indeed, many aptamers internalize upon binding to their protein target on the cell-surface through receptor-mediated endocytosis or, as in the case of the nucleolin-aptamer, macropinocytosis [66]. Thus, they can function as selective delivery vehicles for secondary therapeutic cargos into cancer cells that exclusively express and/or overexpress the aptamer target on their surface. Overall, the development of actively targeted aptamer-based systems will improve methods for delivery of drugs whose therapeutic benefit is limited by instability, lack of specificity or poor bioavailability. So far, different types of aptamer-based drug delivery systems have been devised where, depending on the drug, it is usually intercalated into the aptamer structure or appended to the aptamer sequence through a flexible linker or, otherwise, loaded into diverse formulations of nanocarriers conjugated with aptamers. In such ways, successful delivery of chemotherapeutics [67], toxins [68], therapeutic small interfering RNAs (siRNAs), microRNAs (miRNAs), anti-miRNAs [22, 69] and even large functional RNAs [70] specifically to cancer cells has been achieved by using aptamers as cancer cell-targeting ligands, thus resulting in highly effective therapies. Furthermore, with the aim of overcoming the biological barriers that hinder the efficient and safe delivery of drugs specifically to tumor sites, sophisticated aptamer-based constructs have been designed, such as those allowing therapeutics to reach tumor cells protected by the blood-brain barrier (BBB) [71]. Recently, Macdonald et al. [72], conceived a bifunctional doxorubicin (DOX)-intercalated aptamer, able to bind both to transferrin, highly expressed on the endothelial BBB cell membranes, and to the epithelial cell adhesion molecule (EpCAM) on metastatic TNBC cells. The resulting construct was capable of transcytosing the BBB, upon binding to transferrin and delivering DOX specifically into EpCAM-positive tumor cells, both in an *in vitro* model of BBB and in mouse models of brain metastasis of TNBC.

Using a different approach, our group proved the ability of the anti-platelet-derived growth factor receptor beta (PDGFRβ) Gint4.T aptamer, which we previously generated by GBM cell-SELEX and validated as a high affinity ligand/inhibitor of the receptor in GBM [37, 58] and bone marrow-derived human mesenchymal stem cells [73], to efficiently deliver a drug (Bez 235, PI3K-mTOR inhibitor) to orthotopic (intracranial) GBM xenografts across the BBB. We indeed generated multifunctional nanosystems made up of polymeric NPs, loaded with drug and fluorescent dye, decorated on their surface with the aptamer for both BBB crossing and cancer targeting, exploiting the high expression of PDGFRβ both on BBB endothelial cells and GBM cells [74].

Encapsulating therapeutic agents, in aptamer-decorated nanocarriers offers numerous advantages, including: i) several specific ligands can be linked to the surface of the NPs, increasing the specificity of targeting; ii) multiple drugs can be loaded, allowing for combined therapy; and iii) NPs are very stable, inert and have no toxicity [74, 75].

A recent demonstration of the efficacy of aptamer-based nanoplatforms for hydrophobic drugs’ delivery is given by the elegant study by the Guo et al. [76]. The authors constructed a four-way RNA junction NP for delivering of paclitaxel (PTX), one of the most poorly bioavailable drugs because of its water insolubility and toxicity, with the purpose of maximizing the thermodynamic stability of the construct and the solubility of the PTX cargo. Upon intravenous injections, the construct, equipped with the anti-epidermal growth factor receptor (EGFR) CL4 aptamer [41, 58, 59] as a specific ligand for tumor-targeting, resulted able to drastically inhibit breast cancer growth, with nearly undetectable toxicity and immune responses in mice.

In addition to this, the applications of aptamer-functionalized NPs for cancer therapy appear varied, including their use to remove tumors “surgically”. Indeed, a dynamic magneto nano-therapy using the AS-14 DNA aptamer targeted to mouse Ehrlich’s carcinoma fibronectin was developed to eradicate a tumor in mice by applying an aptamer-guided alternating magnetic field at the single cell level [77].

Moreover, the efficacy of cancer immunotherapy can be improved by nanotechnology and several examples show the potential of nanoparticle-based delivery systems assembled with Abs as active disease-targeting agents to regulate TME and modulate immune response [78, 79]. Similarly, in the last years, aptamers have been proven to act as immunoagents by themselves [80] or upon integration into immunomodulatory nanosystems [81]. Notably, the first CAR-like multivalent aptamer NPs were assembled that could activate T cells and inhibit melanoma growth, opening the way to use aptamer-based constructs for overcoming the safety issues of CAR-T cell therapy [82].

The possibility to directly conjugate aptamers to fluorescent probes and radionuclides makes them exquisite agents for *in vivo* tumor imaging modalities and even theranostic approaches in case the aptamer itself has anti-tumor activities [83]. As we have recently shown, PDGFRβ identifies a subgroup of mesenchymal TNBCs with invasive and stem-like phenotype, and has a crucial role in driving TNBC cell invasiveness and metastases formation [84]. Importantly, due to its inhibitory effect on PDGFRβ activation and downstream signaling pathways, the Gint4.T aptamer was found to be highly effective for both imaging of TNBC xenografts and TNBC cell-derived lung metastases, upon labeling with a near-infrared dye, and for the suppression of metastases formation when administrated intravenously in a mouse model [84]. Aside from direct conjugation of the aptamers to imaging probes, again, aptamer-conjugated NPs can be functionalized with biomedical imaging agents or can be used as containers of imaging and therapeutic agents [85, 86]. Importantly, in both cases the aptamers can achieve comparable or even superior performance than their antibody counterparts [87, 88].

## Aptamers for complementing the use of antibodies

Some recent studies clearly show that the combined use of aptamers and antibodies can be more effective than the monotherapy approach by working in a synergistic or an additive manner. Most of these studies provide support for the clinical evaluation of novel synergistic combinations of immune checkpoint inhibitors and aptamer-based agents for cancer treatment. Ajona et al. [89], tested the anti-tumor efficacy of the combination of an anti-programmed cell death protein 1 (PD-1) mAb and an anti-C5a spiegelmer (a L-stereoisomer aptamer, highly stable because of its non-natural conformation) in preclinical lung cancer models. Interestingly, the aptamer, used to inhibit the signaling of complement C5a with its receptors, revealed only modest antitumor effects as monotherapy but in combination with the antibody, strongly influenced the immune response causing a significant reduction of tumor growth and metastases. Furthermore, Gefen et al. [90], constructed a trimeric version of the T cell immunoglobulin-3 (TIM-3) aptamer, selected by combining a protein- and cell-SELEX protocol, able to delay colon carcinoma cells-derived tumor growth, upon systemic administration to mice as single agent. The blockade of TIM-3 by the currently available TIM-3 antibodies had only little antitumor effect. Importantly, the aptamer was found to be more efficacious than the TIM-3 antibody even when injected at a dose 4.5-fold lower than the antibody. However, as demonstration of the great potential of combining aptamers and antibodies for dual blocking, the aptamer strongly synergized with the PD-1 antibody in prolonging the survival of tumor-bearing mice.

Programmed death-ligand 1 (PD-L1) expression is higher in TNBC than in other breast cancers, providing the rational for recently approved immunotherapy with anti-PD-L1 antibodies, in combination with PTX, for advanced PD-L1-positive TNBC [91]. Currently, enormous effort is devoted to identifying novel actionable biomarkers that may allow combination therapies with immune checkpoint blockade in TNBC. Recently, our group demonstrated the efficacy of a novel combination treatment with the previously validated PDGFRβ aptamer [37, 73, 84] and anti-PD-L1 mAbs in both human and murine *in vitro* settings, consisting of TNBC cells cultured as monolayer or co-cultured with lymphocytes [92]. Importantly, the PDGFRβ aptamer (intravenous administration) potentiated the efficacy of the anti-PD-L1 antibody in inhibiting tumor growth and lung metastasis formation in a syngeneic 4T1 TNBC mouse model by acting on both tumor cells and immune populations. Indeed, by analyzing TME, after the sacrifice of mice, we demonstrated that the combined blockade caused the depletion of FOXP3^+^ Treg cells and the increase of CD8^+^ T cells more consistently than single monotherapies.

Also, we proved that the combined treatment of cancer cells with the highly efficacious anti-EGFR CL4 2’fluoro-pyrimidines RNA aptamer and either an anti-HER2 (Erb-hcAb) antibody [93] or the immunomodulators anti-PD-L1 (10_12 mAb) [93] or anti-CTL4 (ipilimumab) mAbs [94] resulted in more efficient killing of cancer cells than single treatments. These findings provided the rational for generating new bispecific antibody-aptamer conjugates that combine the biological functions of both aptamer and antibody in a single molecule, resulting in increased anti-cancer activity [93, 94]. Specifically, the 5’ amino-terminated CL4 aptamer, conjugated with formylbenzamide formed a stable covalent hydrazone bond with the antibody conjugated with an aromatic hydrazine. The resulting new conjugates acquired increased specificity for double antigen-positive tumor cells, accordingly to the presence of two binding sites in the chimeric construct. When tested on tumor cells, immune cells and tumor and immune cell co-cultures, they inhibited the growth of tumor target cells more efficiently than the parental compounds and, in the case of the EGFR aptamer conjugation with immune-checkpoint mAbs modulators, induced the efficient activation of T cells against cancer cells. Although not yet tested *in vivo* due to the low purification yields of the final constructs, we believe the combination of aptamers and antibodies is a promising approach to potentiate each other’s strengths, overcoming the current therapeutic limitations of both aptamers and antibodies as single agents (Table 1). Indeed, aptamer-based immunoconjugates could have optimal biological features for therapeutic applications such as increased specificity for tumor cells, crosslinking of immune and cancer cells and improved pharmacokinetic and pharmacodynamic properties due to the combined advantages of the small-size aptamer, for increasing tumor penetration, with those of the antibody, which allows a longer half-life in circulation.

Accordingly, a previous study proved that the conjugation of a mAb to an aptamer allows overcoming the therapeutic limitations of aptamers, thus representing a novel approach for rapid, low-cost and high-throughput cancer therapy [53]. Indeed, linking an antibody to the anti-vascular endothelial growth factor (VEGF) pegaptanib aptamer extended the *in vivo* pharmacokinetic of the aptamer without affecting its binding affinity and, on the other hand, the aptamer part of the monospecific immune conjugate penetrated deep into the tumor while anti-VEGF antibody did not.

## Conclusions

An area of intense research is dedicated to finding next generation strategies to efficiently target cancer cells without affecting healthy cells. Cell-SELEX-generated aptamers have many applications (i.e. research reagents, diagnostics, therapeutics and theranostics), thus giving this technology great potential in cancer medicine.

Targeted anti-cancer therapies have recently gained increasing attention in the anti-cancer drug development industry, as these therapies constitute the main branch of precision medicine. The target market for targeted cancer therapies includes drugs used as precision medicine for the treatment of malignant and benign tumors. Numerous cancer targeted therapies have been approved by the FDA to treat various types of cancer and have been commercialized, while many others are still being studied in both clinical trials and preclinical tests.

This market is highly competitive with continuous launches, approvals, and a robust pipeline of new biopharmaceutical products. Nevertheless, the majority of these products show high costs that risk limiting their development. In this view perspective, aptamers may represent a cost-effective alternative, but it is clear that, despite the promising properties, their commercial applications are still limited. Even though thirty years have passed since the first SELEX description and several aptamers have been selected and successful applied in both diagnostic and therapeutic modalities, no aptamers have so far been approved for cancer treatment. However, two aptamers are in clinical trials for cancer therapy: i) AS1411, a DNA quadruplex aptamer against nucleolin, an ubiquitous intranuclear and cytoplasmic phosphoprotein involved in the regulation of cell proliferation, often over-expressed on the surface of tumour cells; and ii) NOX-A12, a Spiegelmer that binds and neutralizes CXCL12 chemokine, which has a crucial role in cell growth and migration [95].

While it is true that there is some reticence about the introduction in the market of new molecules, and that moving from laboratory to clinic typically takes a long time and requires significant financial investment, it is also true that many limitations have kept aptamers from realizing the practical potential that they seemed to have when they were first developed. Major challenges for aptamers practical use cover their discovery process and the strategies for improving their binding affinities for diagnostic applications, and therapeutic efficacy, half-life and stability for patients’ treatment. Nevertheless, researchers worldwide are working hard to overcome those obstacles. Specialized partitioning methodology, e.g., capillary electrophoresis-based techniques make possible to select aptamers even by a single-round selection [96]. In addition, by integrating the SELEX technology with high-throughput sequencing technology allows identifying the best candidate aptamers in early selection cycles thus strongly reducing times and costs of the process [23]. Further, the types of chemical modifications for adapting aptamers to any desired applications is growing fast [97]. Efficacious approaches have been developed for engineering aptamers toward their use as diagnostics [98], *in vivo* imaging agents [99], therapeutics [100] and delivery agents [67, 97]. Notably, the global aptamers market was estimated at 32 million of dollars in 2018 and is expected to reach 100 million of dollars in 2025, with a compound annual growth rate (CAGR) of 15.5% [101].

Thus, it is possible to envisage that in the near future, in the era of combination therapy, we will fight cancer by attacking it from multiple flanks by complementing existing drugs with novel oligonucleotide aptamer-based therapeutics, overcoming drug resistance and increasing therapeutic efficacy.

## Abbreviations

BBB: blood-brain barrier
DOX: doxorubicin
EGFR: epidermal growth factor receptor
EpCAM: epithelial cell adhesion molecule
GBM: glioblastoma
HER2: human epidermal growth factor receptor 2
mAbs: monoclonal antibodies
NK: natural killer
NPs: nanoparticles
PD-1: programmed cell death protein 1
PDGFRβ: platelet-derived growth factor receptor beta
PD-L1: programmed death-ligand 1
PEG: polyethylene glycol
PTX: paclitaxel
SELEX: systematic evolution of ligands by exponential enrichment
TIM-3: T cell immunoglobulin-3
TME: tumor microenvironment
TNBC: triple-negative breast cancer
VEGF: vascular endothelial growth factor

## Declarations

### Copyright

© The Author(s) 2021.
